# American Mammals Susceptibility to Dengue According to Geographical, Environmental, and Phylogenetic Distances

**DOI:** 10.3389/fvets.2021.604560

**Published:** 2021-03-10

**Authors:** Ángel L. Robles-Fernández, Diego Santiago-Alarcon, Andrés Lira-Noriega

**Affiliations:** ^1^Facultad de Física, Universidad Veracruzana, Xalapa, Mexico; ^2^Red de Biología y Conservación de Vertebrados, Instituto de Ecología, Xalapa, Mexico; ^3^CONACYT Research Fellow, Red de Estudios Molecualres Avanzados, Instituto de Ecología, Xalapa, Mexico

**Keywords:** biotic interactions, DENV 1-4, machine learning, random forest, risk assessment, sylvatic cycle, wild reservoir

## Abstract

Many human emergent and re-emergent diseases have a sylvatic cycle. Yet, little effort has been put into discovering and modeling the wild mammal reservoirs of dengue (DENV), particularly in the Americas. Here, we show a species-level susceptibility prediction to dengue of wild mammals in the Americas as a function of the three most important biodiversity dimensions (ecological, geographical, and phylogenetic spaces), using machine learning protocols. Model predictions showed that different species of bats would be highly susceptible to DENV infections, where susceptibility mostly depended on phylogenetic relationships among hosts and their environmental requirement. Mammal species predicted as highly susceptible coincide with sets of species that have been reported infected in field studies, but it also suggests other species that have not been previously considered or that have been captured in low numbers. Also, the environment (i.e., the distance between the species' optima in bioclimatic dimensions) in combination with geographic and phylogenetic distance is highly relevant in predicting susceptibility to DENV in wild mammals. Our results agree with previous modeling efforts indicating that temperature is an important factor determining DENV transmission, and provide novel insights regarding other relevant factors and the importance of considering wild reservoirs. This modeling framework will aid in the identification of potential DENV reservoirs for future surveillance efforts.

## 1. Introduction

Human impacts on natural environments have altered the structure and dynamics of ecological interactions [e.g., (1)], opening opportunities for a dramatic increase of novel emergent diseases (2, 3). Given that viruses represent the main pathogenic group from where new emergent and re-emergent diseases affecting human populations come from, many efforts are directed toward viruses infecting mammals [e.g., (4)]. Recent studies showed that emergent infectious disease events in human populations are more likely in regions with warmer and humid climates, places where land use change is directed toward agricultural systems and large urban areas, and in areas with higher mammal diversity (particularly rodents, bats, and non-human primates) (3–7). A disease that has increase its incidence in tropical regions due to human factors is the dengue virus (DENV), which is a mosquito-borne disease endemic to tropical areas and that has a sylvatic transmission cycle infecting bats and non-human primate species [e.g., (8, 9)]. The natural sylvatic cycle has been altered due to anthropogenic activities, leading to an increase in its incidence for example by global warming that favors the geographic expansion of its mosquito vector [e.g., (10); but see (11) for a case where the dengue season is projected to be shorter due to the negative impact of climate change on the life span of the Asian tiger mosquito *Aedes albopictus*, an introduced species, into different cities of the USA]. Thus, in order to be able to predict and prevent DENV expansion and increases in incidence, it is necessary to understand the ecological and biogeographic components of its sylvatic cycle (12, 13), particularly in the case of the American continent where the sylvatic enzootic cycle is poorly understood.

Studies on DENV have demonstrated that a large amount of variation in the incidence is explained by environmental variables such as temperature and precipitation, but there are no straightforward ways to generalize transmission dynamics given the complex interactions among climatic variables and local ecological factors [e.g., (13–15)]. Furthermore, characteristics of a geographical range such as size and overlap [e.g., (4, 16)], and the richness of both vectors and host vertebrates are important determinants of a pathogen host breadth (7). In the case of DENV, the geographic expansion of one of its mosquito vectors (e.g., the invasive tiger mosquito) into places with immunologically naïve populations poses a major risk (10, 17), particularly because researchers do not know ahead of time what species will enter the sylvatic cycle of the disease [e.g., (9)]. Thus, we need to develop more effective and preventive alternatives to reduce the risk of potential hazards becoming a real problem, and we also need to take the perspective of wild organisms and how they are affected by human activities [i.e., OneHealth or EcoHealth; (18, 19)]. Ultimately we want to predict if the sylvatic cycle of DENV is geographically close to important human population sites, and how likely DENV is to switch among wildlife, domestic animals, and humans [e.g., (5)].

DENV is a pan-tropical self-limiting disease with a human death toll estimated in 10,000 fatalities and 100 million symptomatic cases per year (15). It is caused by a single-stranded RNA virus belonging to the *Flavivirus* genus, it is transmitted by different mosquito species (Diptera: Culicidae), and belongs to a viral family (Flaviviridae) that includes other major diseases such as yellow fever (YFV), West Nile (WNV), and Zika viruses (10). DENV includes at least four genetically different serotypes or clades, which are embedded in human urban cycles (mostly transmission among humans via mosquitoes of the genus *Aedes*, in particular *Aedes aegypti*) and also in ecologically complex sylvatic cycles (9, 10). This viral disease is original from the Old World, evidence indicates that the four serotypes emerged from an ancestral virus via a transmission cycle involving non-human primates and mosquitoes about a 1,000 years ago (20). The Old World origin of the dengue virus has been confirmed by genetic studies, and historical records suggest the arrival of the virus during the slave trade from Africa (21, 22). Dengue was introduced into the Americas starting in the 1600s in some Caribbean Islands and by the 1690s reached the continent via Panama (22). The four dengue serotypes have dramatically extended their geographical range and incidence across the Americas since 1943 (20), establishing endemic-epidemic cycles in all the tropical and some sub-tropical regions of the continent (22, 23). Regarding sylvatic cycles, there are only two places where transmission cycles are reasonably well-determined, one is in Senegal and the other is in Malaysia where non-human primates belong to a permanent sylvatic cycle (12, 24). Although sylvatic cycles have not been recognized in the Americas, the four human serotypes have been identified infecting wild mammals other than non-human primates (i.e., rodents, bats, marsupials) both in urban and non-urban areas (25, 26). Interestingly, molecular studies have identified sequence divergence of the four viral strains circulating in wild mammals compared to those circulating in human populations (25), which suggests that some adaptation to wild hosts has already happened. Yet, researchers modeling expansion of DENVs have done it via geographic projections of *Aedes* spp. vectors [e.g., (27, 28)], and little effort has been invested in determining the vertebrate mammal assemblages potentially maintaining DENV sylvatic cycles in the Americas. Moreover, every human DENV serotype has a sylvatic ancestor (29) and well-known mammal Orders (i.e., primates, rodents and bats) that are reservoirs for many viral families are readily infected with DENV in Latin America. Thus, many wild mammal species may belong to the enzootic sylvatic cycle of DENV in the Americas (9, 25, 26), and it is a medical and veterinary priority to discover who those species are.

The use of analytical statistical tools can take advantage of knowledge on host breadth, environmental variables, geographic distributions, and host phylogenies to develop predictive frameworks to generate geographical assessments of infection risk, which can be applied to either host assemblages or a focal host species by either a group of pathogens or by a specific pathogen of interest [e.g., (30)]. Thus, our aim is to predict sylvatic host assemblages of DENV in the Americas via machine learning statistical protocols, in order to create preventive frameworks useful for health agencies (e.g., CDC) that can aid to forecast the probability or risk of potential hazards (31). Here, we analyze the relevance of geographic, environmental, and phylogenetic distances among potential wild host species of DENV to predict their susceptibility to the virus. This predictive framework allows to detect host assemblages or species with higher risk of infection across these three biodiversity dimensions, as well as to identify potential host species where the pathogen has not been previously detected or that have not been previously considered in field screening projects. This approach will help to direct surveillance and field efforts providing cost-effective decisions on where to invest limited resources to untangle DENV sylvatic cycles in the Americas, in case they are already established.

## 2. Materials and Methods

### 2.1. Host—Parasite Data

We did an exhaustive review of information about the incidence of dengue virus in mammals. We started from the information available in (32), from which we obtained the references and subsequently obtained information from 15 articles, and complemented with spatial information from DBatVir (http://www.mgc.ac.cn/DBatVir/) (33), although these were comparatively fewer points. This search allowed us to collect a total of 249 incidences in 30 species of mammals distributed geographically in the Americas and is available as an open database (34). Since only 10 species of mammals contained 80% of the information, we decided to use this as the initial susceptibility cutoff threshold to conduct model training (Table 1). Thus, model predictions will be consistent with the environmental, geographic and phylogenetic information from this set of species.

**Table 1:** List of top 10 highest DENV incidence species according to raw data.

**Order**	**Family**	**Species**	**Incidence**
Chiroptera	Phyllostomidae	*Artibeus jamaicensis*	66
Primates	Atelidae	*Alouatta caraya*	48
Chiroptera	Phyllostomidae	*Glossophaga soricina*	16
Didelphimorphia	Didelphidae	*Marmosa murina*	15
Chiroptera	Phyllostomidae	*Artibeus planirostris*	14
Didelphimorphia	Didelphidae	*Didelphis marsupialis*	12
Chiroptera	Molossidae	*Molossus rufus*	10
Chiroptera	Phyllostomidae	*Artibeus lituratus*	9
Chiroptera	Phyllostomidae	*Desmodus rotundus*	8
Chiroptera	Vespertilionidae	*Myotis nigricans*	7

### 2.2. Input Data Processing

We estimated the susceptibility to DENV based on species' distances from the geographic, environmental and phylogenetic spaces. To calculate the geographic distance matrix between pairs of species, we calculated the geographic distance between the centroids of the largest polygon for each mammal species in the Americas available from the International Union for Conservation of Nature polygons (International Union for Conservation of Nature 2020; the IUCN Red List of Threatened Species, version 2018-2. https://www.iucnredlist.org. Downloaded on 05 January 2020). A total of 1778 species of land mammals were selected for America. We carried out this geographical data manipulation with the R package sf (35).

Then, for between-species environmental distance, we estimated the distance between the maxima of the probability density with a Gaussian kernel associated with the first two principal components from the 19 bioclimatic layers from the WorldClim database [approximately 80% of the total variance explained; (36)]. The probability density was based on a sample of 10,000 points contained within the geographic range of the species based on the IUCN polygons mentioned above. In this case, the maxima of the probability density were considered as a surrogate of the ecological niche centroid for each species. The principal components were based on a correlation matrix on layers with a 2.5 min spatial resolution; the use of these layers was based on the idea of using a low dimensionality of environmental variables while accounting for most of the information. For phylogenetic distances, first we generated a pruned phylogenetic tree with the species of terrestrial mammals of the American continent, which we then used to calculate the phylogenetic distances between pairs of species with the ape R package (37). The original phylogenetic tree was from (38). All of the above information was summarized in a database with the environmental, geographic and phylogenetic information among all pairs of species (≈3,000,000 rows). Next, we took the average of one species with respect to all others in order to summarize the information about the three distances (i.e., geographic, environmental, and phylogenetic). This rendered a table of 1,778 species × 3 distances.

### 2.3. Machine Learning Modeling

For each host-parasite assemblage, we created a data set taking each *n* host species and the phylogenetic, environmental, and geographic distances calculated above. Based on this data set, we selected the species considered in the pathogen incidence cutoff (see above) and labeled these species as susceptible. Subsequently, we took a random sample of species outside of the cutoff set, balancing the sample size with respect to the cutoff set and labeling these species as unknown. This generated a data set with two susceptibility classes (susceptible and unknown) and three independent variables (environmental, geographic, and phylogenetic distances). To this data set, we also added the interactions between the independent variables and separated the data set into two sets to train the models and validate them (70–30%). Following Kuhn (39), we generated a set of random forest models optimizing their parameters with a modeling grid (40) and with 10 times 10-fold cross validation for each sample. With the optimal model we regressed the entire data set and obtained the probability of susceptibility given the distances and their interactions, also calculating the importance of each variable. Each time we classified the species as susceptible with a standard probability threshold [i.e., *p*(*x*) > 0.5]. We repeated this procedure 1,000 times, each with a different sample, in order to look for convergence in the probability calculation, as well as its uncertainty. We then estimated the mean probability of susceptibility for each host species in each host-pathogen assemblage, along with its standard error (41).

### 2.4. Susceptibility Geographical Richness

In order to provide a spatial pattern of susceptibility to dengue across American mammals, we assigned the average susceptibility probability of each host species to each of their geographic ranges. Subsequently, we selected only species with probability of susceptibility *p*(*x*) > 0.5 and considered these as susceptible. Finally, the geographic ranges of susceptible species were used to generate a species richness map of potential dengue hosts on the geographic space. This was done using the raster (42) and fasterize (43) R packages.

### 2.5. Statistical Validation of Spatial Patterns

To test our model on geographic space, we placed empirical validation points of where dengue has been found in the field and applied a statistical hypothesis on the spatial distribution of the punctual empirical pattern with respect to our susceptibility richness map. We tested as null hypothesis (*H*_0_) that the density of empirical pathogen points is not a function of the richness of susceptible host species, and as an alternative hypothesis (*H*_*a*_) that the density of empirical pathogen points depends on the richness of susceptible host species according to the random forest model. We performed this test using the likelihood ratio test (LRT) for each hypothesis, getting in all cases *p* < 0.05 and rejecting *H*_0_ in all cases. We implemented this analysis with the R package spatstats (44, 45).

### 2.6. Mapping Susceptibility as a Phylogenetic Continuous Trait

We used contMap and fastAnc functions from phytools R package (46) to use the predicted DENV susceptibility on each species as a trait and map it on the phylogeny. This might be even more relevant given that the phylogenetic distance was a very important variable from the overall machine learning modeling protocol.

## 3. Results

**Figure 1:**
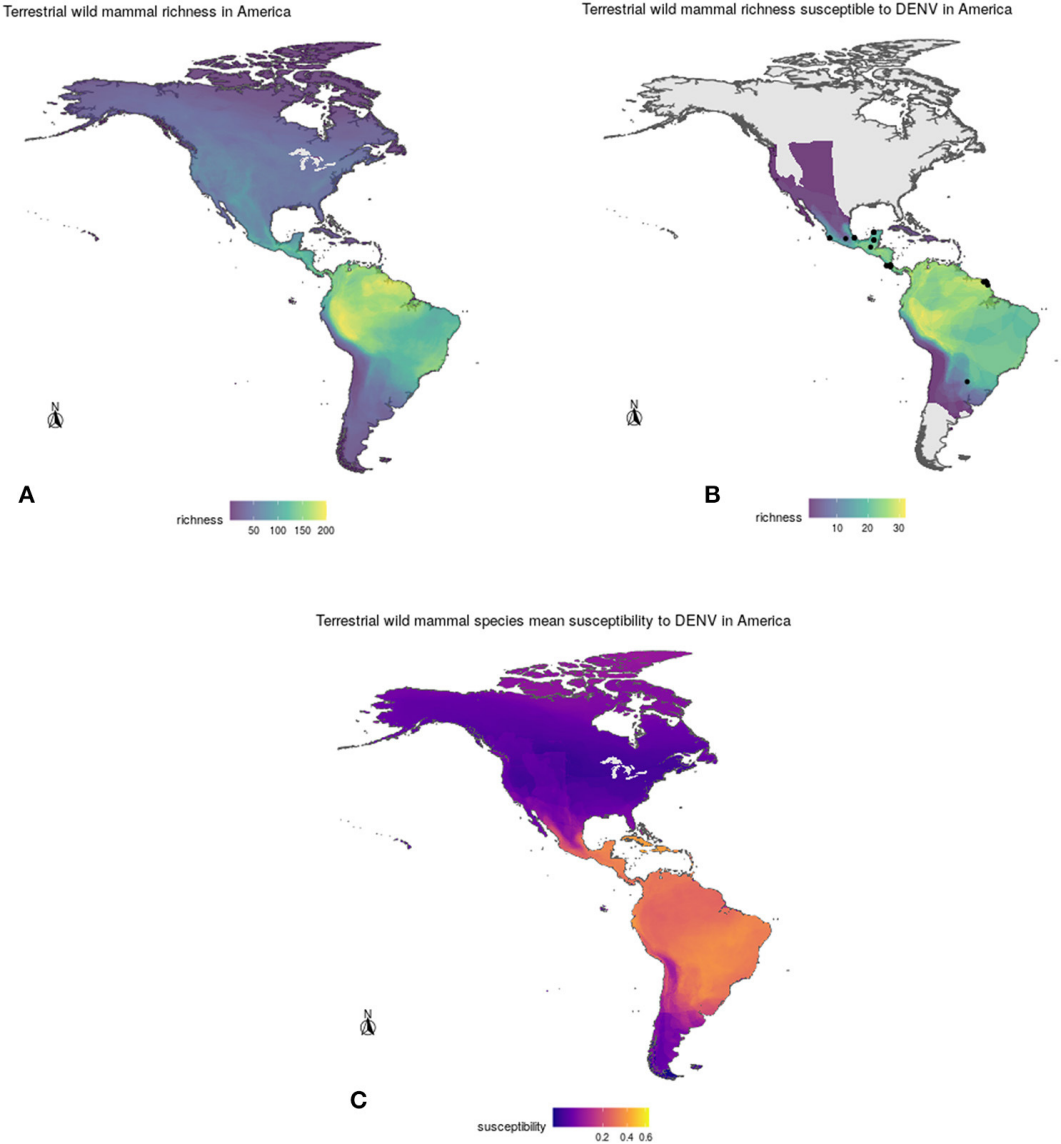
**(A)** Geographic distribution of total mammal species richness (**top left**), **(B)** richness of DENV susceptible species (**top right**), and **(C)** mean susceptibility to DENV (**bottom**) of wild mammals in the Americas according to model projections. See section 2 for details.

**Figure 2:**
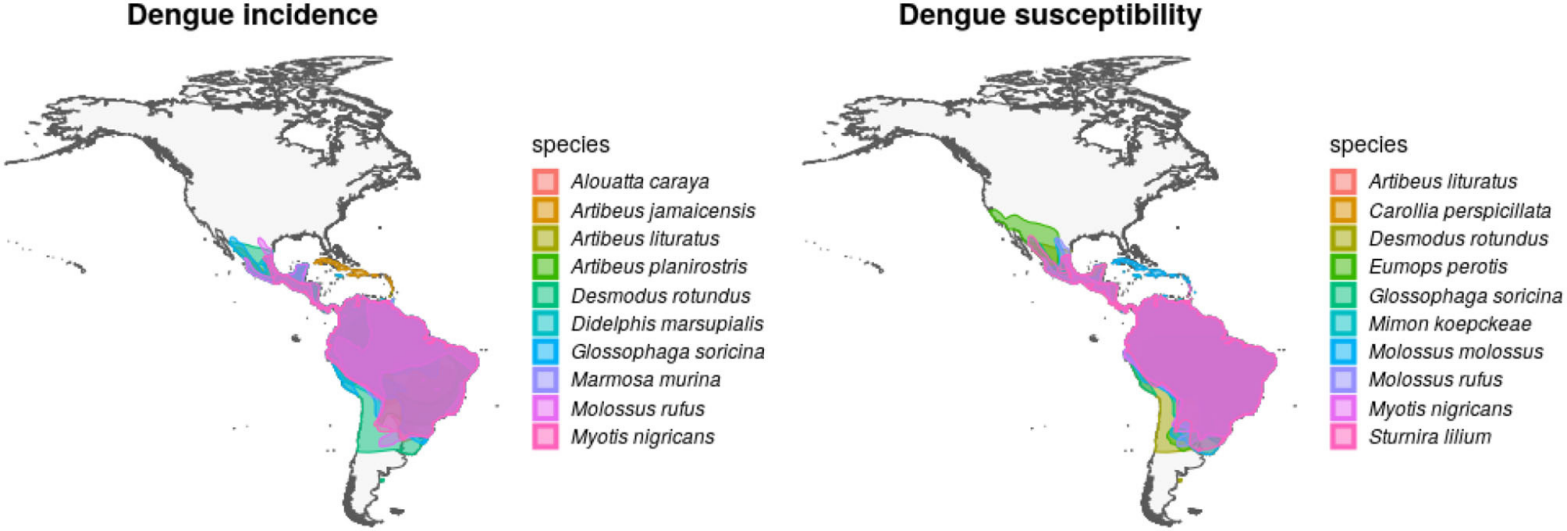
Geographic distribution of mammal species with high incidence and susceptibility to DENV. **Left** panel shows the top 10 wild mammal species with highest DENV incidence according to published information. **Right** panel shows the top 10 most DENV susceptible wild mammal species according to the random forest models.

**Figure 3:**
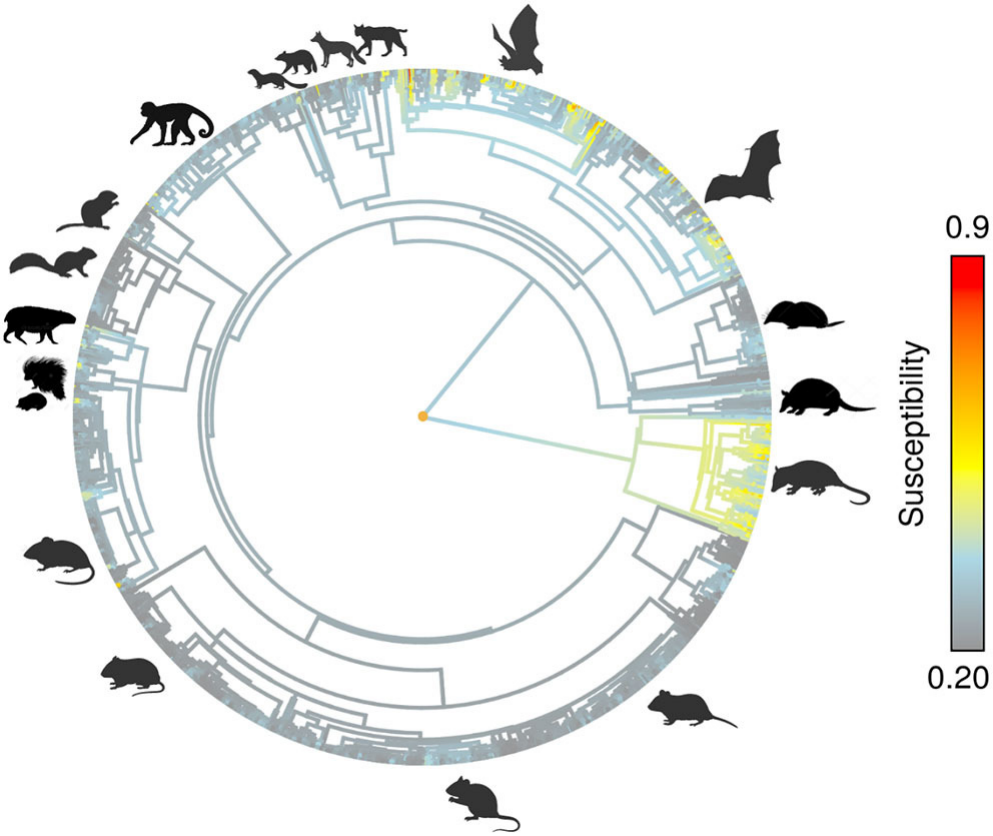
Predicted susceptibility as continuous trait on mammals phylogeny.

**Figure 4:**
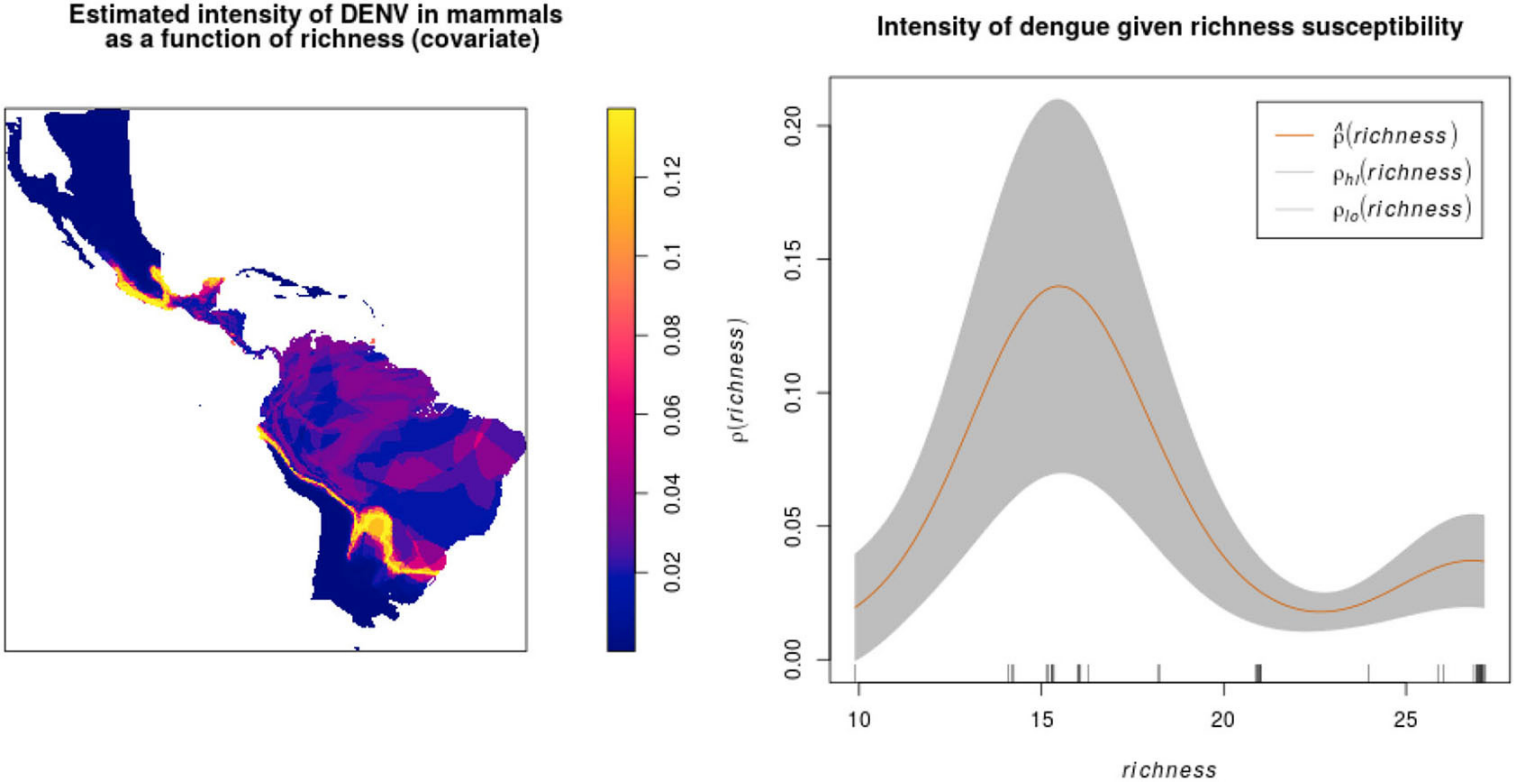
Estimated DENV intensity among American wild mammals given susceptible species richness as a covariate. The plot shows $\hat{\rho }\left(richness\right)$ against covariate values of richness, together with 95% confidence bands (between ρ_*lo*_ and ρ_*hi*_) assuming a non-homogeneous Poisson point process.

After overlapping the ranges of 60 susceptible species [i.e., species with probability of susceptibility *p*(*x*)>0.5], the species richness map for susceptible species to DENVs showed higher values in tropical America and decreasing values toward southern South America and western North America (Figure 1). This pattern was statistically corroborated by independent localities with incidence of DENV in mammals, indicating that it follows a spatial point pattern depending on species richness predicted as susceptible, with the highest density of DENV in mammals predicted at a species richness close to 20 (Figure 1B). The mean susceptibility to DENV from the 1,774 mammal species evaluated followed a similar geographic trend with respect to susceptible species richness (Figure 1B). The top 10 species with highest susceptibility to dengue are shown in Figure 2 and their associated susceptibility score in Table 2. These belong to bats of different trophic guilds, but the species at the top of the list is the common vampire bat that feeds primarily on mammalian blood (Table 2). High values of predicted susceptibility, however, are not exclusive of bats; when mapped as a continuous character on the mammals' phylogeny, high susceptibility appears in different groups such as marsupials (Figure 3). Interestingly, the pattern of DENV intensity given richness of susceptible species showed two regions with high values (Figure 4). One appears in central Mexico in the northern limit of the Neotropical biogeographic region, in what looks like a belt that connects the Gulf of Mexico and the Pacific coast across continental Mexico, and an isolated portion at the north of the Yucatan Peninsula. The second region of high intensity appears in South America, also in what looks like a belt that connects the southern portion of Brazil to northern Peru (Figure 4). It is interesting to compare the richness of all mammals and that of susceptible species, which are roughly coincident, at least for areas of highest species richness (Figure 1).

**Table 2:** List of top 10 most DENV susceptible species according to the random forest models.

**Order**	**Family**	**Species**	**Susceptibility**
Chiroptera	Phyllostomidae	*Desmodus rotundus*	0.909
Chiroptera	Phyllostomidae	*Artibeus libratus*	0.898
Chiroptera	Phyllostomidae	*Glossophaga soricina*	0.877
Chiroptera	Phyllostomidae	*Sturnira lilium*	0.875
Chiroptera	Molossidae	*Eumops perotis*	0.850
Chiroptera	Molossidae	*Molossus rufus*	0.845
Chiroptera	Molossidae	*Molossus molossus*	0.834
Chiroptera	Vespertilionidae	*Myotis nigricans*	0.821
Chiroptera	Phyllostomidae	*Mimon koepckeae*	0.821
Chiroptera	Phyllostomidae	*Carollia perspicillata*	0.814

**Figure 5:**
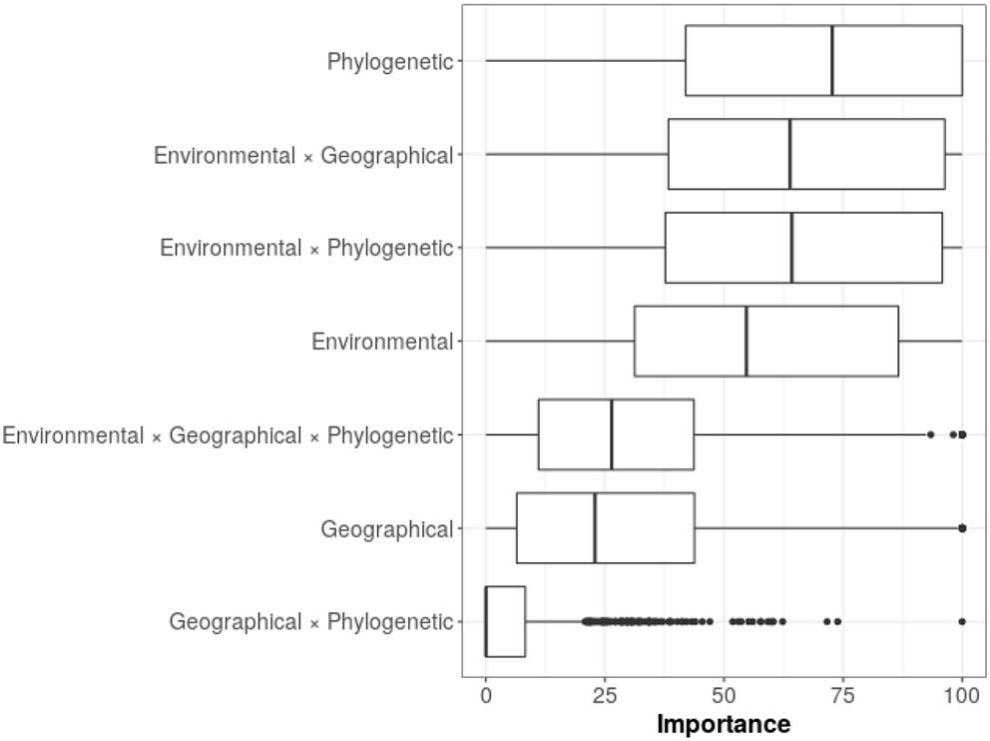
Variable importance from random forest models. The mean percentage values for the variable importance are as follows: phylogenetic = 67.5; environmental*geographical = 62.6; environmental*phylogenetic = 62.3; environmental = 55.8; environmental*geographical*phylogenetic = 29.9; geographical = 28.5; geographical*phylogenetic = 6.38.

The variable importance for all the models, indicated three most important predictors: the phylogenetic distance between species, the interaction between environmental and geographical distances, and the interaction between environmental and phylogenetic distances (Figure 5). These predictors were followed in importance by environmental distance, while the rest of the predictors and their interactions appeared with lower importance. Although we observed that models had very large variances regarding variable importance, phylogenetic distance remained in the first place of importance.

**Figure 6:**
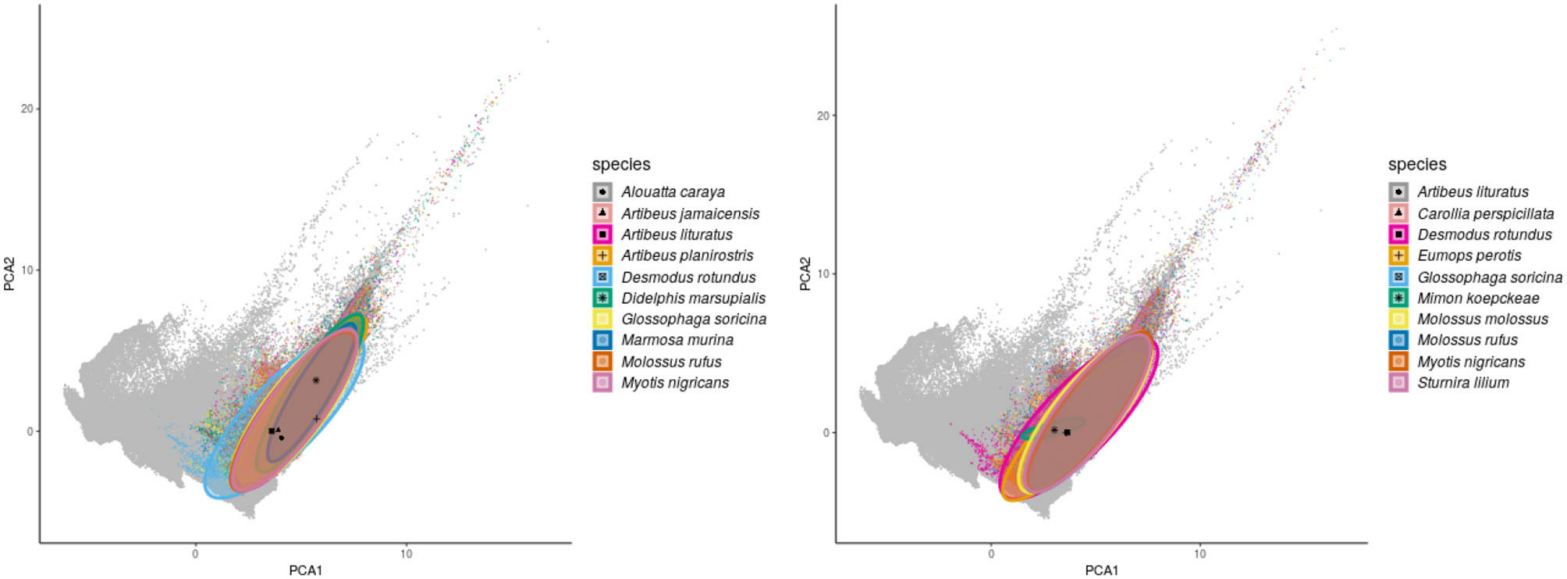
Environmental ellipsoids with centroids. **Left** panel shows the top 10 wild mammal species with highest DENV incidence according to published information. **Right** panel shows the top 10 wild mammal species most susceptible according to random forest models.

**Figure 7:**
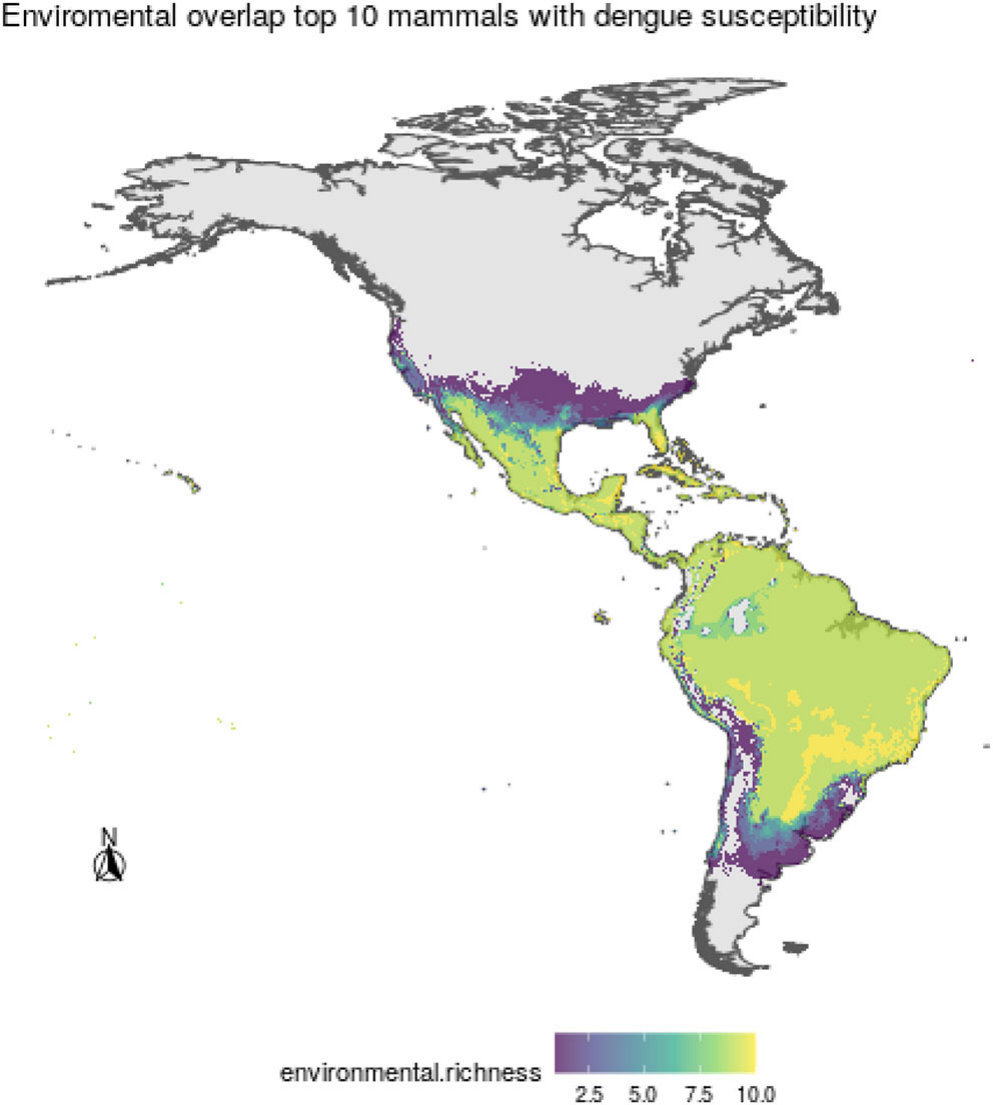
Areas of environmental overlap for the top 10 most susceptible wild mammal species to DENV, where higher values indicate more coincidence among species ecological niches.

Regarding the environmental space, mammal species of high incidence and high predicted susceptibility largely overlap in their niche requirements across the Americas (Figure 6). These correspond mostly to warm regions and semiarid to wet areas. Although it is something we had not formally tested [e.g., following ecological niche background similarity tests; (47)], the ecological niches of these two sets of species showed high amount of overlap according to the ellipsoidal models that summarize their ecological niches (Figure 6). The areas with the highest amount of suitable conditions are mostly in tropical America, with extreme values in South America in southern Amazonia, northern Chaco, and south of the Eastern Highlands ecoregions, as well as the West Indies and Florida regions, including some parts of Mexico across the Yucatan Peninsula and parts of the eastern slope toward the Gulf of Mexico (Figure 7).

## 4. Discussion

Researchers have largely overlooked the empirical and theoretical (i.e., model predictions) study of wild reservoir assemblages of DENV. In part this may be due to the great challenge imposed by the difficulty of discovering competent hosts, while we recognize that finding new non-human hosts is also desirable. Here, we show a species-level susceptibility prediction to dengue in wild mammals of the Americas as a function of three biodiversity dimensions (ecological, geographical, and phylogenetic spaces).

Model predictions coincide with already known geographical distribution patterns for this disease in tropical and subtropical America, where most vulnerable species are bat species of different trophic guilds. Predictions of susceptibility to dengue in wild mammals appears to be highly influenced by phylogenetic distance among species, and also by the environment (i.e., distance between the species' optima in bioclimatic dimensions) in combination with geographic distance.

To our surprise, most of the empirical and modeling studies of DENV are focused on the urban cycle of transmission (17). Although this is expected given the economic and human health impacts of DENV in urban areas, we suggest that current model projections may fail in the long term if they disregard the role that wild reservoirs (and their phylogenetic, environmental, and geographical limiting factors) may play in the ecological transmission of DENV. Wild reservoirs are mostly unknown in the Americas and yet every DENV affecting humans has a sylvatic ancestor in the Old World (21, 29). Considering that the DENV1-4 serotypes circulating in wild mammals in the Americas already have some sequence divergence compared to the same serotypes infecting humans (25), it would be fair to say that the establishment of a DENV sylvatic cycle in the Americas is only a matter of time; also opening the opportunity for the development of novel DENV strains adapted to wild mammals. In this sense, our modeling exercise complements all ecological and biogeographic models of previous DENV by providing the perspective from the sylvatic assemblage of potential wild mammal hosts, which may act as an effective ecological reservoir (48). This effort will become even more relevant in the near future, given that anthropogenic activities are bringing closer wild animal assemblages to human populations (49).

The most comprehensive and recent global geographic projection of DENV took into account climatic variables, human population and economic factors, and the distributions of *Ae. aegypti* and *Ae. albopictus* vectors (15). Such models continue to predict previously identified tropical and subtropical areas as suitable for DENV, where temperature (68% of variation), precipitation (13%) and relative humidity (6%) are the main environmental variables affecting transmission in that order. Moreover, an estimated average of 3.8 billion people (>50% of the global population) live in areas suitable for DENV transmission particularly in Asia (15). Models predict that for 2050 a large portion of southeastern USA, northern areas of Argentina and higher elevation areas of central Mexico will be suitable for DENV transmission. Some other areas such as East Africa will observe DENV declines due to lower suitability because of hotter and drier conditions (15); it is well-known that temperatures higher than 35 degrees Celsius reduce DENV transmission due to reduced mosquito survival (50).

The main DENV vector in urban areas is *Ae. aegypti*, which is well-adapted to human dwellings and possesses population characteristics that makes it ideal to maintain DENV infection cycles [e.g., insecticide resistant, desiccation resistance, multiple probing for blood meals on different individuals, capable of using heterogeneous sources for laying eggs avoiding competition and crowded locations; (10)]. Temperature is considered the main climatic factor affecting positively the expansion of DENV across the world, particularly to areas currently free of this virus [e.g., temperate areas of North America and Europe; (15, 50)]. *Aedes albopictus*, one of the most invasive mosquito species worldwide, is suggested to be a competent vector of DENV, and under some situation is even better than *Ae. aegypti*, and what may be limiting its vectorial capacity seems to be more related to its ecology and to its biological attributes (50).

The global expansion of the invasive tiger mosquito (*Ae. albopictus*) may aid in DENV geographic expansion. *Aedes albopcitus* has higher tolerance to lower temperatures compared to *Ae. aegypti*, and also inhabits peri-urban and sub-urban areas feeding in a larger array of vertebrate hosts, making it ideal as a bridge vector across the human-domestic-wildlife interface (17). Furthermore, the establishment of DENV in wild reservoirs may aid in the expansion and invasion success of this pathogen. Different species of bats have been positive to different DENV serotypes across the world; in the Americas the four DENV serotypes were detected in French Guiana in a sample that included bats, rodents, and marsupials (25). In Ecuador and Costa Rica, bats inhabiting urban areas were reported as exposed (seroconverted) to the 1, 2, and 3 DENV serotypes (51). In Mexico, DENV-2 serotype has been reported in at least three species of bats in both intact rainforests and disturbed forests in areas of the Yucatan Peninsula (9); however, in a survey of more than 1,900 individuals of different species of rodents and bats on the Pacific slope of Mexico there were no positive results for DENV, which was probably due to the spatiotemporal heterogeneity of transmission (52). Furthermore, empirical evidence of non-human primates able to act as reservoirs of epidemic DENV [in particular DENV-2 in an animal breeding facility in the Philippines; (8)] highlights the need to survey potential wild animal reservoirs both in urban (e.g., zoos) and non-urban (e.g., peri-urban forest fragments, agricultural lands) areas, where spillback of urban DENV from humans toward non-human primates or other wild animals can happen [e.g., (53)]. Finally, in a recent analysis within the Flaviviridae, DENV was the only generalist virus capable of infecting several species across the Chiroptera, Rodentia, and Didelphimorphia (32). Therefore, we suggest that researchers around the world must consider a priority the inclusion of the wild community of potential reservoirs within their DENV surveillance efforts, particularly in areas where the interactions between humans and wild animals is higher (e.g., agricultural lands, peri-urban areas).

Our results confirm that Chiroptera contains a highly susceptible set of species to DENV. In addition, the geographic location of species recorded with DENV from different mammal orders (including those mentioned above; also see table of incidences from this work) confirmed the spatial prediction of our models. This pattern is apparently due to evolutionary and ecological processes as indicated by the importance of phylogenetic and environmental distances in generating these predictions. Even more, when we use the predicted susceptibility as a continuous trait and map it on the phylogeny (see Figure 3), it is evident that other groups rather than Chiroptera may be highly susceptible to DENV. As past research highlights, phylogenetic distance is one of the most important factors to predict the likelihood of pathogen transmission in hosts of several taxonomic groups not related to mammals [e.g., (54)]. It is possible that this same process is modulating DENV interaction with mammals. At the same time, however, this should be modulated by species environmental tolerances (i.e., ecological niches) and their spatial context. In fact, this research suggest that highest susceptibility to DENV in American mammals could be constrained not only phylogenetically (as incidence and susceptibility prediction indicate) but also environmentally and geographically. Most species of DENV susceptible mammals appear to share similar environmental requirements (some with wide tolerances, specially in the precipitation axis) and show different degrees of geographic overlap in their distribution areas mostly along the tropical region.

The findings from this biogeographical approach should be put in perspective when DENV-associated reservoirs and vectors are analyzed. Here we highlight the value of combining their phylogenetic, environmental and distributional contexts in order to provide a first effort toward a better understanding of DENVs ecological dynamics in non-urban settings. We hope that these results and methodological approach aids in a solid proposal both to confirm and discover new DENV hosts in the community of wild mammals, while at the same time suggesting what are the main drivers behind DENV transmission.

## Data Availability Statement

The datasets analyzed for this study can be found in the Zenodo repository [https://doi.org/10.5281/zenodo.4018028].

## Author Contributions

ÁR-F conducted the analyses. All the authors contributed in the data collection, evaluation of the results, and writing of the manuscript in its different sections.

## Conflict of Interest

The authors declare that the research was conducted in the absence of any commercial or financial relationships that could be construed as a potential conflict of interest.
